# Selective Attention Increases Choice Certainty in Human Decision Making

**DOI:** 10.1371/journal.pone.0041136

**Published:** 2012-07-16

**Authors:** Leopold Zizlsperger, Thomas Sauvigny, Thomas Haarmeier

**Affiliations:** 1 Department of Neurology, RWTH Aachen University, Aachen, Germany; 2 Department of General Neurology, University of Tübingen, Tübingen, Germany; University of Muenster, Germany

## Abstract

Choice certainty is a probabilistic estimate of past performance and expected outcome. In perceptual decisions the degree of confidence correlates closely with choice accuracy and reaction times, suggesting an intimate relationship to objective performance. Here we show that spatial and feature-based attention increase human subjects' certainty more than accuracy in visual motion discrimination tasks. Our findings demonstrate for the first time a dissociation of choice accuracy and certainty with a significantly stronger influence of voluntary top-down attention on subjective performance measures than on objective performance. These results reveal a so far unknown mechanism of the selection process implemented by attention and suggest a unique biological valence of choice certainty beyond a faithful reflection of the decision process.

## Introduction

Life boils down to a series of choices you make or do not, many of them depend on pending outcomes of previous choices. Choice certainty – a probabilistic estimate of past performance and expected outcome [1], [2], [3] – therefore has a strong impact on how decisions are formed and enacted [4]. Asked for your certainty in a decision – you can readily answer, it is as intuitive as actually making the decision in the first place. Recent neuronal recordings in behaving monkeys [1] and rats [2] confirm the notion that in every perceptual decision not only a choice is made, but also an evaluation of the quality of evidence that added to the decision is generated. A single population of monkey lateral intraparietal area (LIP) neurons was found to represent both choice and certainty in a motion discrimination task using post-decision wagering [1]. A computation of choice certainty along with the choice itself is consistent with the close correlation of the degree of confidence with decision accuracy [5] and reaction times [6], objective performance metrics similarly influenced by selective attention [7], [8], [9], [10]. Given that neural activity in LIP describes temporal, feature-based [11] as well as spatial dynamics [12] of attention, and selective attention optimizes probabilistic inference under uncertainty [13] and alters phenomenological appearance [14], [15], [16], it is surprising that precise effects of selective attention on choice certainty have been rarely addressed [17], [18]. We show for the first time that spatial and feature-based attention increase human subjects' certainty more than accuracy in visual motion discrimination tasks. Both for certainty reported via post-decision wagering [1], [19] and numerical confidence ratings, we observe higher changes of overall confidence levels than in actual performance. This dissociation of subjective and objective performance measures suggests a unique biological valence of choice certainty beyond a faithful reflection of the decision process and rather proposes an implementation by different mechanisms, differing neuronal substrates or in a larger neuronal network.

## Methods

### Subjects

41 healthy subjects, 24 females and 17 males with a mean age of 25±4 years participated in this psychophysical study. All subjects had normal or corrected-to-normal visual acuity and normal color vision as assessed by standard Ishihara plates. Written informed consent was obtained from all subjects according to the Declaration of Helsinki and the guidelines of the local ethics committee of the faculty of medicine of the University of Tübingen, which approved the procedures.

### Stimulus presentation

Subjects viewed all stimuli binocularly from a distance of 55 cm on a 19′′ TFT-display (native resolution 1280×1024 pixels) driven by a Linux computer running the nrec visual stimulation, data acquisition and experiment control software package (http://nrec.neurologie.uni-tuebingen.de, created by F. Bunjes, J. Gukelberger et. al.) at a refresh rate of 60 Hz in a dark, quiet room.

### Stimuli

To explore objective and subjective performance with and without selective attention, we designed four precue – postcue experiments. Subjects were instructed to stringently follow a selective attention cue preceding the stimulus; spatial attention was applied in two of the experiments, feature-based attention in the two others. Participants always had to discriminate the global direction of one of two motion stimuli presented simultaneously and differing in location (spatial attention tasks) or color (feature attention tasks). They could not respond before a postcue indicated which of the two stimuli they actually had to evaluate. Both a decision and the confidence in it was given trial by trial, all subjects followed the instructions as illustrated by changes of objective performance with attention.

**Figure 1:**
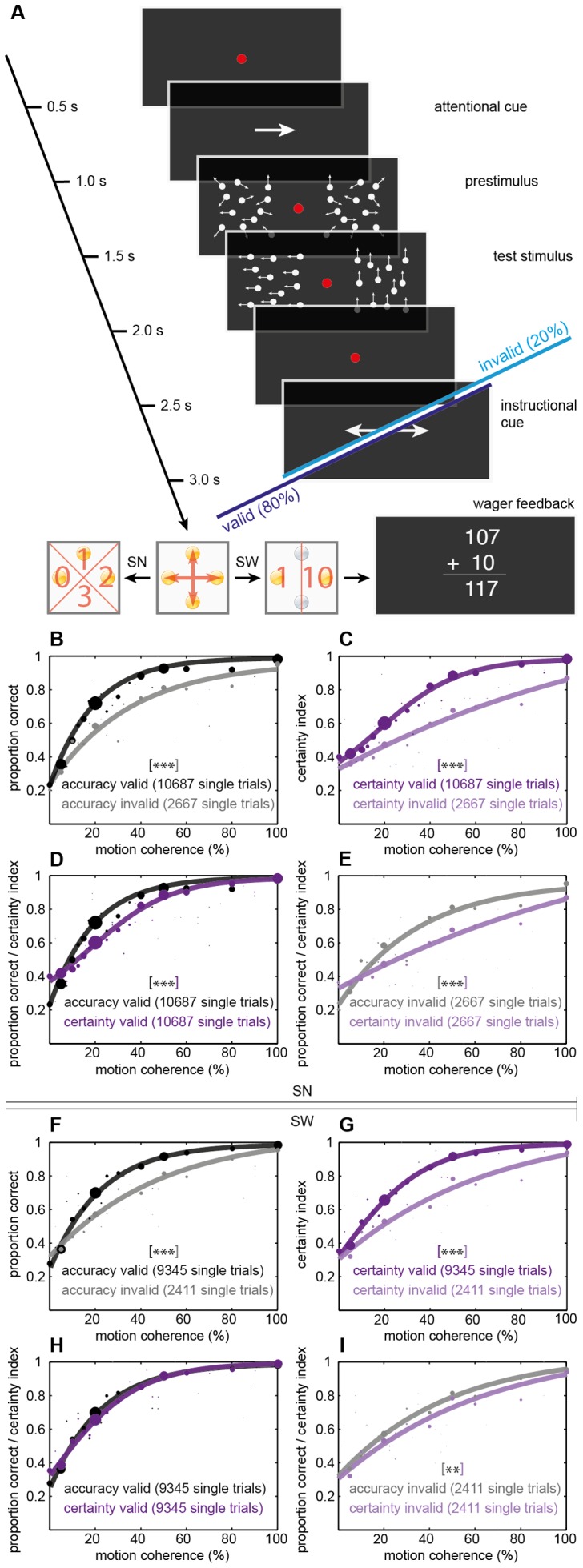
Spatial attention: behavioral tasks and effects of attention on accuracy and certainty. **A**, Timing of events for an example spatial attention trial. The test stimulus consisted of two RDKs presented simultaneously left and right of the fixation point (1.5 – 2s), level of motion coherence and direction of global motion (four alternatives) were modulated on a trial-by-trial basis. An arrow before stimulus presentation (0.5 – 1s) indicated which RDK covertly shift attention to, a second arrow after the stimulus (2.5 – 3s) instructed subjects which RDK they actually had to indicate the direction of coherent motion for. Valid cueing - as defined by congruent orientation of the attentional and the instructional cue - was applied in 80% of trials, see dark blue option. Invalid cueing (incongruent arrows, light blue option) was applied in the remaining 20% of trials. Subjects reported perceived motion direction with a first button press and decision certainty with either a second press of the same buttons using four predefined numerical ratings (SN) or of two of the buttons corresponding to a high (10) or low (1) wager (SW). For the separate wagering variation, wager feedback was given via a continuously updated point score adding or subtracting the chosen virtual bet. **B–I**, Percentage of correct responses or certainty index, respectively, vs. motion coherence for all subjects. Data points show the proportion of correct choices or the certainty index, respectively. Size of the points is scaled pursuant to the number of corresponding trials. Solid curves are logistic fits to the data using a Maximum Likelihood criterion. ** tags p<0.01; *** p<0.001 derived from model comparison statistics using Monte-Carlo simulations of the two respective fits, missing asterisk in h: no significant difference between fits. Spatial attention with numerical certainty ratings: B–E; spatial attention with certainty wager: F–I. B,F compare accuracy for valid and invalid cues. C,G compare certainty for valid and invalid cues. D,H compare accuracy and certainty for valid trials. E,I compare accuracy and certainty for invalid trials.

The visual stimulus in the **spatial attention experiment** consisted of six periods, each lasting 500 ms (see Fig. 1A). After a first fixation period (central fixation dot, red, diameter 2 arcmin) a central arrow (dimensions: 3°×1°, white, luminance 384 cd/m^2^) instructed subjects to covertly shift attention either to the left or right hemifield. This attentional cue was followed by two random dot kinematograms (RDKs) each of which covered a square of 9°×9° and was centered 13° right and left, respectively, of the fixation point. Each RDK consisted of 475 white squares (side length  = 0.8 arcmin, lifetime  = 500 ms, dot density ∼6 dots/deg^2^, luminance 384 cd/m^2^) on a black background (luminance 0.14 cd/m^2^), all moving incoherently, that is, in all possible directions with a resolution of 1°, at a common speed of 6 deg/s. After the presentation of this first pair of RDKs (*prestimulus*), a second pair of RDKs, the test stimulus, started [20], [21]. The properties of the test stimulus were identical to those described for the prestimulus except that a certain percentage of the dot elements moved coherently in the same direction (either up, right, down or left). The percentage of coherently moving dots in an individual trial was chosen equally either from four predefined steps (5%, 20%, 50%, or 100% of all dots) or according to an adaptive staircase procedure [22]. Start level of this procedure was 80% coherence. The change in coherence started with a step of 20% and step size was halved in case the last change had caused convergence toward the 62.5% correct threshold, step size was doubled (if possible) in case the change had led further away from this threshold. The staircase procedure was terminated and started new when the step size had reached a value of 0.02%. Motion coherence was always identical for the two RDKs in a given trial, global motion direction could be the same or different as randomly chosen by the computer. After a subsequent second fixation period, a second arrow indicated for which of the two RDKs subjects had to indicate the direction of coherent motion (four-alternative forced choice). Valid cueing – as defined by congruent orientation of the precue and the postcue – was applied in 80% of trials. In the first variation of this task (Fig. 1A, *SN*) subjects reported their post-decision certainty by pressing one of four buttons used to signal the perceived motion direction right before. Uniformly, subjects were asked to rate how confident they were in their decision on a scale of 0 – 3 (*0* being “I guessed”, *3* “I was sure”, *1* and *2* equally spaced steps of the continuum in between), distributing their responses over the available scaling. 13 of the subjects performed this task, each one completing between 1000 and 1100 single trials.

In the second variation of this task (Fig. 1A, *SW*) post-decisional certainty was indicated by means of post-decision wagering [19]. Subjects were instructed to place a wager of either 1 or 10 virtual points by pressing the left (1) or right (10) button on the interface device after their direction choice. They would win or lose this amount depending on whether their first choice was correct or incorrect. Subjects started with an amount of 100 virtual points. Immediate feedback was given after the second button press by showing a central counter (dimensions: 7.5°×6°, white, luminance 384 cd/m^2^) for 1000 ms. 11 of the subjects performed this task, each one completing between 1000 and 1100 single trials.

**Figure 2:**
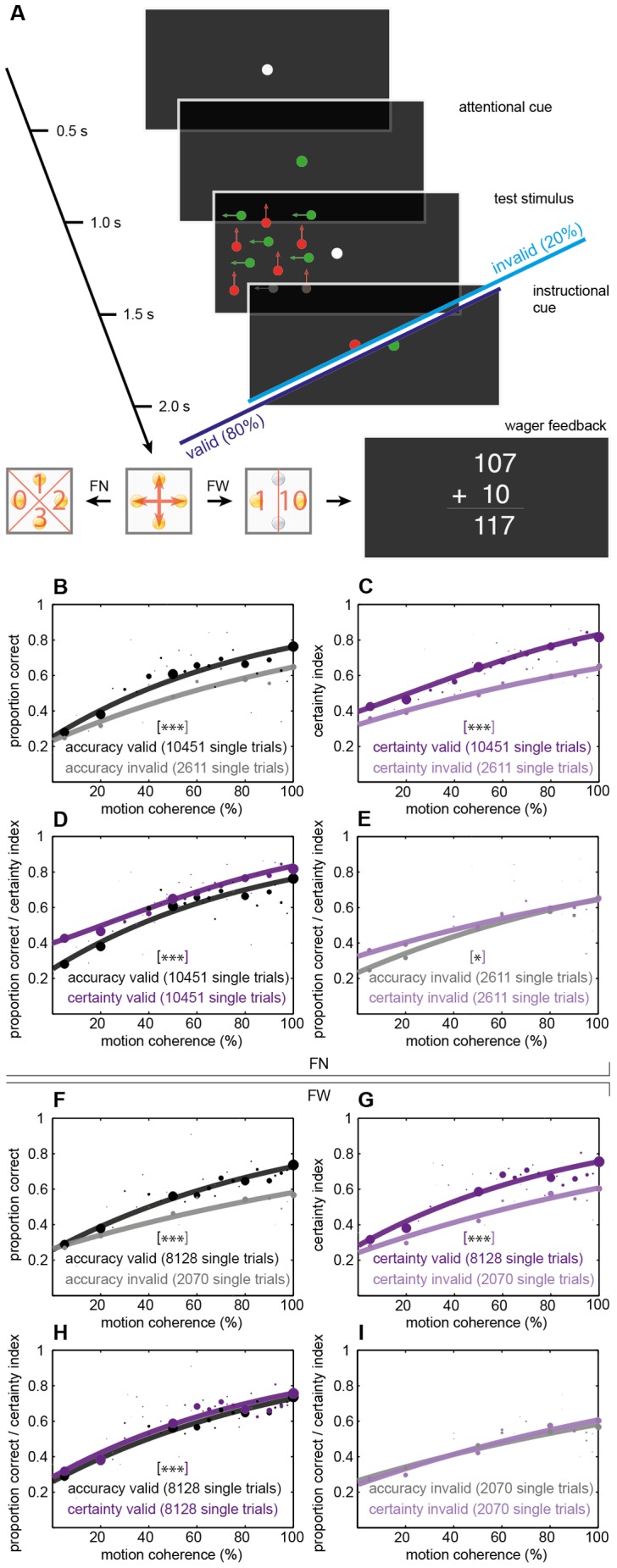
Feature-based attention: behavioral tasks and effects of attention on accuracy and certainty. **A**, Timing of events for an example feature-based attention trial. The test stimulus consisted of two interlacing RDKs differing in color and was presented randomly left or right of the fixation point (1–1.5 s), modulation of the independent RDKs otherwise matched the spatial attention tasks. Color changes of the fixation point to red or green before (0.5–1 s) and after the presentation (1.5–2 s) of this test stimulus instructed subjects which dot elements to direct attention to and which to actually indicate the direction of global motion for. The other cueing and response modalities were identical to the spatial attention tasks. **B–I**, Percentage of correct responses or certainty index, respectively, vs. motion coherence for all subjects. Conventions are identical to the spatial attention tasks in Fig. 1. * tags p<0.05; *** p<0.001 derived from model comparison statistics using Monte-Carlo simulations of the two respective fits, missing asterisk in I: no significant difference between fits. Feature-based attention with numerical certainty ratings (FN): B–E, feature-based attention with certainty wager (FW): F–I. B, F compare accuracy for valid and invalid cues. C, G compare certainty for valid and invalid cues. D, H compare accuracy and certainty for valid trials. E, I compare accuracy and certainty for invalid trials.

The visual stimulus in the **feature-based attention experiment** consisted of four periods, each lasting 500 ms (see Fig. 2A). After a first fixation period (central fixation dot, white, diameter 2 arcmin, luminance 384 cd/m^2^) a color change of the fixation point to red or green instructed subjects which dot elements to direct attention to in the subsequent RDK. This RDK (*test stimulus*) covered a square of 9°×9° and was randomly centered 13° right or left of the fixation point. It consisted of 235 red and 235 green squares (side length  = 0.6 arcmin, lifetime  =  ∼100 ms, dot density ∼6 dots/deg^2^ on a black background (luminance 0.14 cd/m^2^)). Luminance of red and green dots was set equal, however, without strictly controlling for isoluminance. Both groups of dot elements were implemented as independent RDKs with otherwise identical experimental settings as in the spatial attention tasks. During test stimulus presentation the fixation point turned white, to change color again subsequently to red or green instructing subjects which of the dot elements subjects had to report the direction of coherent motion for (four-alternative forced choice). Valid cueing – as defined by matching colors of the precue and the postcue – was applied in 80% of trials. Subjects signaled post-decision certainty as per the variations of the experimental design applied in the two spatial attention tasks. In the numerical certainty variation (Fig. 2A, *FN*) 11 of the subjects completed between 1100 and 1200 single trials each, in the wagering variation (Fig. 2A, *FW*) 11 of the subjects completed between 900 and 1000 single trials each.

For all variants, an initial practice run of 50–70 trials familiarized the subjects with the procedure and stabilized psychophysical thresholds, these trials were not used for further analysis. Subjects had seven seconds to complete both responses, a new trial started right after the second button press or after point score presentation.

### Eye movement recordings

During all experiments, eye movements were monitored using a custom built video system taking the pupil's center as measure of eye position. Recordings were stored at a sampling rate of 50 Hz and quality of fixation was analyzed offline. In particular, deviations from the fixation point (eye position) were examined for the period of test stimulus presentation. Under all experimental configurations our subjects maintained stable fixation as indicated by horizontal (h) and vertical (v) eye positions close to 0° and overall small standard deviations: SN h:0.81°±1.04°, v:0.47°±0.99°; SW h:0.17°±1.73°, v:0.47°±1.87°; FN h:0.42°±1.38°, v:0.14°±1.15°; FW h:0.28°±1.15°, v:0.28°±1.13°.

### Data analysis

The psychometric functions of choice accuracy and certainty were plotted for valid and invalid cueing separately as proportion of correct decisions, high wagers or certainty ratings, respectively, against the coherence level of the motion signal. To match the scaling and allow for a direct comparison of accuracy and certainty data, low wagers were assigned a value of 0.25 (matching chance level), high wagers of 1 (matching perfect discrimination). Consistent with the instructions on how to use the numerical certainty ratings, *0*/left button was coded 0.25; *1*/up button 0.50; *2*/right button 0.75; *3*/down button 1. To test for significant influences of attention on accuracy and certainty and also for differences between the two measures within a given attention condition, the data were fitted by logistic functions using a Maximum Likelihood criterion. Transformed likelihood ratios of respective pairs of fits – a first fit based on the data of both conditions and a second one treating the two conditions separately – were taken to be significantly different if exceeding 95% of transformed likelihood ratios obtained through Monte Carlo simulations (10000 simulations were performed in each comparison). Logistic fits and their statistical comparisons were performed using routines of the Palamedes [23] toolbox for Matlab, for further specifics of the model definition and statistical simulations including source code see the reference documentation of *PAL_PFLR_ModelComparison*.

**Figure 3:**
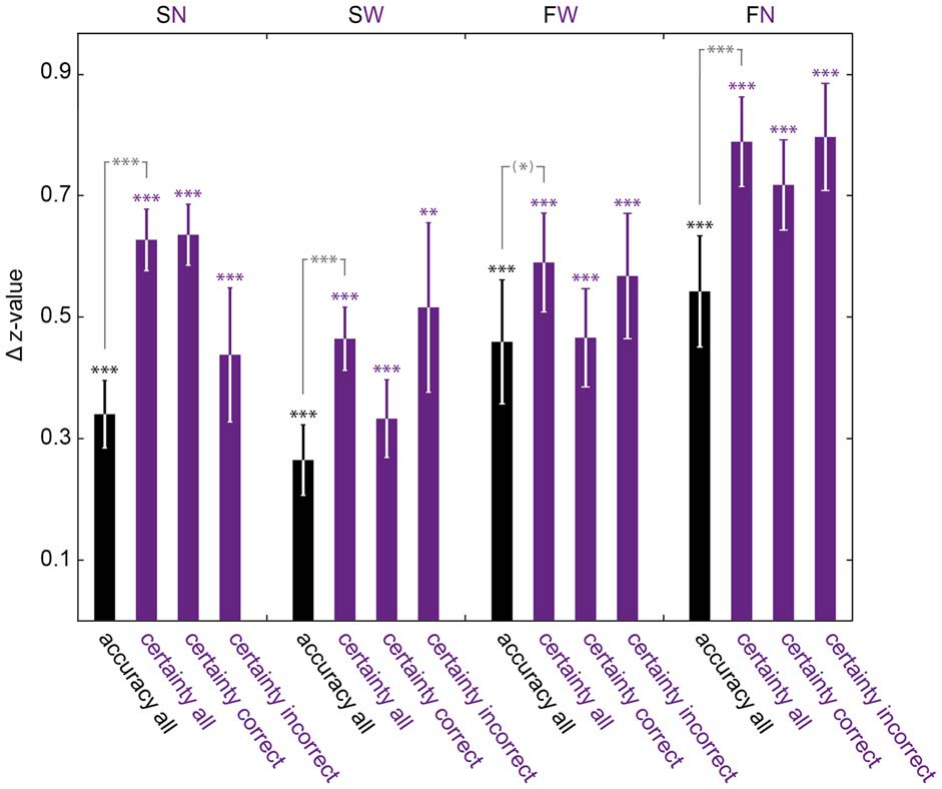
Attention-related change in z-standardized accuracy and certainty. Differences of mean performance values across subjects observed for the valid condition minus those observed for the invalid condition plotted as Δ z-value (means ± standard errors of the mean). Positive *Δ*s reflect increases with attention. Both z-transformed accuracy and certainty increase significantly with attention for all four experiments. Certainty increases with valid cueing when correct and incorrect trials are analyzed separately. Increases of certainty are significantly larger than increases of accuracy (grey labels) for SN, SW and FN and tend to be in FW. Detailed p-values see main text. (*) tags p<0.1; ** p<0.01; *** p<0.001 derived from paired *t*-tests.

To quantify and further investigate attention-related variation, every subject's mean accuracy and certainty of coherence levels with 90 and more trials each for valid and invalid cues were z-transformed to a common mean (0) and standard deviation (1). Standardized accuracy measures for valid and invalid cueing were subjected to paired t-tests, as were standardized certainty measures. Beyond that, certainty data were analyzed and plotted for correct and incorrect choices separately in Fig. 3. Paired differences of valid and invalid accuracy measures (Δ z-values) were compared to corresponding certainty deltas in paired t-tests to examine the effects of attention on both objective and subjective performance.

## Results

To investigate the effects of top-down selective attention on choice accuracy and certainty in perceptual decision making, we had human subjects discriminate global visual motion embedded in noise for attended and unattended stimuli and rate the confidence of their response. Variations of a random dot stimulus we used before [20], [21] allowed selective cueing of spatial or feature-based attention in separate experiments. Subjects rated their post-decision certainty on a trial-by-trial basis either by numerical certainty ratings [5] or an intuitive post-decision wagering procedure [19], in which subjects are offered immediate feedback via a constantly updated point score.

### Spatial attention increases choice certainty

During each trial of the spatial attention tasks illustrated in Fig. 1A, observers maintained fixation while two random dot kinematograms (RDKs) were briefly presented right and left of a central fixation point. An arrow appearing before the RDKs instructed subjects to covertly shift attention to one of the two hemifields. A second arrow after dotfield presentation instructed subjects which of the two RDKs they had to indicate the direction of coherent motion for. Valid cueing as defined by congruent orientation of the attentional and the instructional cue was applied in 80% of trials. Invalid cues (20% of the trials) let us analyze motion discrimination for stimuli not covered by attention.

Psychometric data were plotted as percentage of correct responses or certainty index, respectively, vs. motion coherence in Fig. 1B–I and fitted by logistic functions. Reflecting the dependency of task difficulty on motion coherence, discrimination performance increased for higher motion coherences in both attention conditions and for the two post-decision certainty variations as expected [21]. We found higher sensitivity to visual motion for valid cueing [21], [24] as indicated by a leftward shift of the psychometric accuracy function with attention (Fig. 1B, F). That is, for a given coherence level attention increased the proportion of correct choices. This improvement in performance for an attended stimulus is the signature of attentional modulation on behavior [25], [26] and demonstrates that subjects reliably followed the cue instructions. Both for numerical ratings and post-decision wagering, certainty similarly increased with higher motion coherence (Fig. 1C, G). Leftward shifts of the respective psychometric functions with attention were evident as well and even more pronounced than for choice accuracy. Juxtaposing psychometric accuracy and certainty fits for valid (SN: Fig. 1D; SW: Fig. 1H) and invalid cueing (SN: Fig. 1E; SW: Fig. 1I) separately showed a decreasing dissociation of the asymptotic functions in the valid from the invalid condition. This indicates an even stronger effect of selective attention on post-decision certainty as compared to choice accuracy. Accordingly, model comparison statistics using Monte-Carlo simulations revealed highly significant increases with attention in accuracy and certainty (SN, SW: all p<0.001). Within the invalid cueing condition accuracy and certainty were significantly different (SN: p<0.001; SW: p = 0.005; Fig. 1E, I), within the valid condition they were for SN (p<0.001, Fig. 1D) and were not for SW (p = 0.223, Fig. 1H). Significantly differing logistic fits of accuracy and certainty by model comparison statistics in the invalid (Fig. 1I) as opposed to the valid condition (Fig. 1H) in SW further highlight the dissimilar effects of selective attention on accuracy and certainty.

While equalizing lowest certainty to 0.25 and highest to 1 reflects both the stochastics of our four-alternative choice task and parallels the subjects' instructions, a certainty-metric fit might well be created by assigning different arithmetic values. To avoid specific conceptual and mathematical preconditions fit comparisons rely on, the underlying data were brought to a common scale via z-standardization in a second, independent statistical approach. The subjects' proportion of correct decisions and mean certainty for equidistant numerical ratings (SN) or wagering (SW), respectively, were z-transformed to a common mean (0) and standard deviation (1). The differences of values observed for the valid condition minus those observed for the invalid condition were calculated and plotted as *Δ z-value* in Fig. 3. Positive Δs of accuracy data (black bars in Fig. 3) reflected significant increases in objective performance with attention for both spatial attention tasks (paired *t*-tests; SN, SW both p<0.001), just as positive Δs of certainty data (purple bars) did for subjective performance (SN, SW both p<0.001). By analyzing certainty for correct and incorrect choices separately, we found attention-related increases of post-decision confidence regardless of whether choices were correct or not (Fig. 3, correct: SN, SW both p<0.001; incorrect: SN p<0.001, SW p = 0.001). The attention-related difference in certainty for pools of trials not differing with respect to objective performance suggests that the increase in certainty with attention was not a sole reflection of the increase in choice accuracy. Does selective attention indeed differ in its effects on subjective and objective performance? Δ z-values for accuracy and certainty were compared in a final paired *t*-test: z-standardized certainty increased significantly more with selective attention than accuracy (SN, SW both p<0.001; labeled grey in Fig. 3). This mirrors the stronger leftward shifts with attention seen for the psychometric functions of certainty as compared to accuracy and demonstrates specific effects of selective attention on subjective performance measures not explained by changes in objective performance.

**Figure 4:**
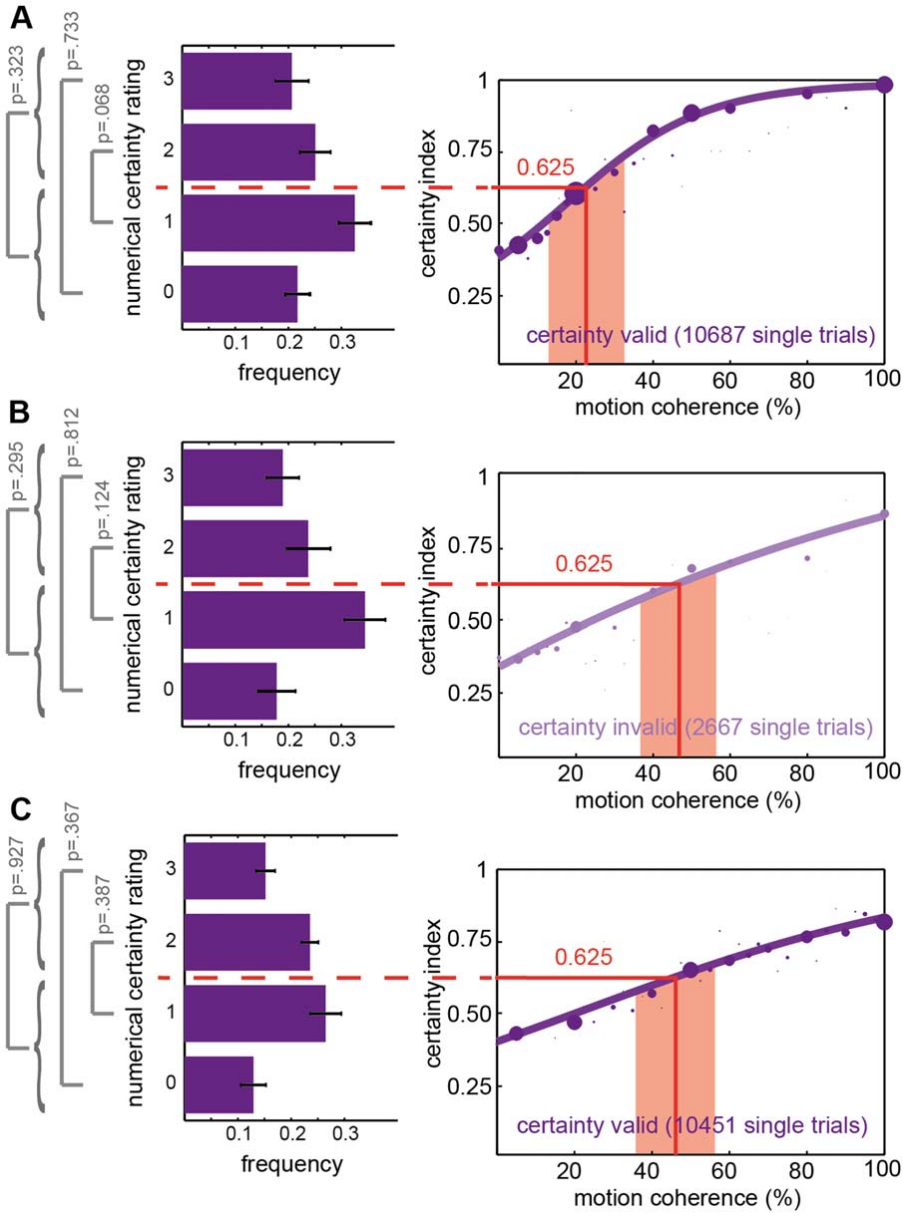
Observers use high and low certainty ratings uniformly. Frequencies of respective certainty ratings for a ±10% range of coherence levels around the inflection point of the individual logistic fits: **A**, valid trials in the spatial attention task with numerical certainty. **B**, invalid trials in the spatial attention task with numerical certainty. **C**, valid trials in the feature-based attention task with numerical certainty. Right: Certainty index as function of motion coherence, group data (part of Fig. 1C/2C). Left: Means and S.E.M.s of the frequencies of the four different certainty ratings for all subjects. Grey labels: P-values, statistical significance of paired *t*-tests comparing the frequency of certainty ratings on a group level. Numerical certainty ratings in the left and right panel: *0* corresponds to 0.25, *1* to 0.5, *2* to 0.75, *3* to 1.

Further statistical and experimental controls were performed to substantiate these conclusions. Please note that z-statistics are independent of the specific numerical certainty measures entering transformation only as long as the different categories are equidistant. For instance, the categories in the numerical certainty experiments could have been assigned the numbers 1, 2, 3, and 4 without affecting the results of the z-statistics. Values 0.25, 0.5, 0.75, and 1.0 were chosen for better comparability to the accuracy space, reflecting the stochastics of the task and subjects' instructions. While equidistance was the necessary consequence of the two options in the wager experiment, it was the most parsimonious presumption in the experiment offering four options: If certainty categories are equally spaced and all subjects use the four equidistant levels uniformly, at the inflection point of the logistic fit to the certainty data the observers are expected to apply the lowest as frequently as the highest rating – and similarly for the interjacent pair of ratings. Consequentially, the two lowest levels should be chosen as often as the two highest categories. We observe this exact behavior for numerical confidence ratings: as illustrated in Fig. 4, we determined the frequency of each certainty rating for a constant interval of coherence levels ranging from 10% below to 10% above each observer's certainty threshold (Fig. 4A–C: light red area). In our four-alternative forced choice paradigms, this threshold is the coherence level corresponding to a certainty index of 0.625, equaling the point of inflection. Comparing the subjects' frequencies of applying the lowest (*0*) vs. the highest rating (*3*) in the spatial attention task, there is no significant difference between groups (paired *t*-test; valid-cue-trials p = 0.733, see Fig. 4A; invalid-cue-trials p = 0.812, Fig. 4B), as there is none for the two middle categories (*1* vs. *2*; valid p = 0.068, invalid p = 0.124) or the two lower vs. the two higher categories (*0* and *1* combined vs. *2* and *3* combined; valid p = 0.323, invalid p = 0.295). Albeit uncorrected for multiple comparisons, none of the *t*-tests reaches statistical significance, indicating an equal distribution of low and high certainty ratings, i.e. a Gaussian-like distribution of choice categories.

Next, a few exceptionally strong shifts in post-decision certainty might have biased group statistics, so accuracy and certainty measures were controlled for outliers via modified z-scores using the median of the absolute deviation about the median as an outlier resistant estimator in place of the standard deviation in z-score calculations. Modified z-scores have not been used for statistical significance tests, but rather as a numerical guide to identifying outliers. For SN ∼1.5%, for SW ∼3.0% of mean accuracy-certainty pairs per coherence level exceeded a modified z-score of 3.5, i.e. the standard criterium defining outliers [27] and were excluded from a rerun of the z-statitistics. No p-value mentioned above changed, except for the Δ z-values accuracy vs. certainty in SW from p<0.001 to p = 0.003.

Since the direction of global motion in both RDKs was random and motion coherence was always identical for a given trial, in about a fourth of the trials both dotfields contained the same signal. To test if a quarter of the invalid trials being effectively valid had distorted the results, both model comparison and z-statistics were repeated, this time confined to the 75% of trials involving only non-congruent motion signals. Correction for congruent trials did not affect the significance of the effects reported here.

Furthermore, as performance measures saturate out asymptotically at higher coherence levels in the spatial attention tasks, we repeated the z-statistics restricted to coherences less than 50%. By contrasting objective and subjective measures along the main slope of the psychometric functions we excluded that different ceiling effects may account for the observed dissociation of performance measures with attention. Z-standardized certainty increased significantly more with selective attention than accuracy for these motion coherences in both versions of the spatial attention task (SN p = 0.003; SW p = 0.002).

**Figure 5:**
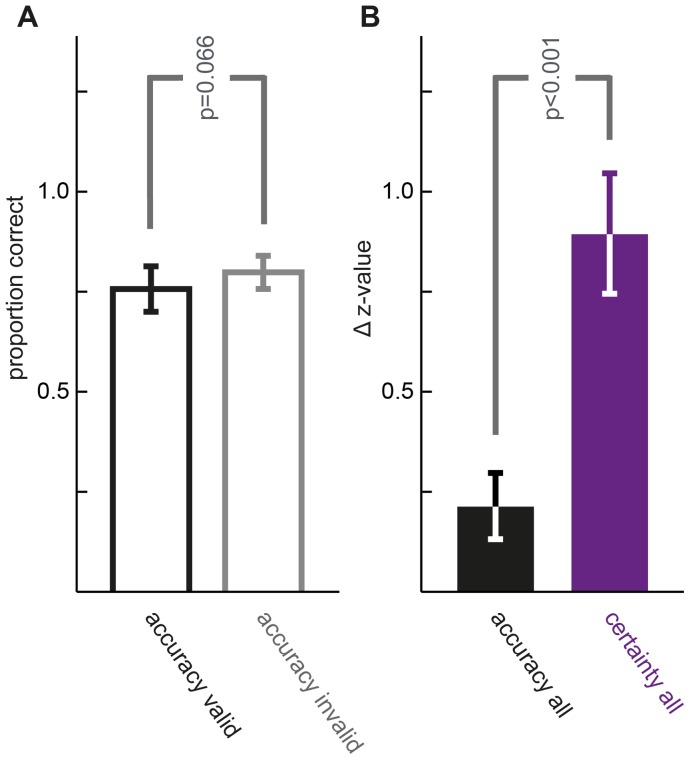
Z-standardized certainty increases significantly more with attention than z-standardized accuracy if controlled for the overall success rate. **A**, Mean accuracy for the group of valid cue trials next to the mean accuracy for invalid cues, all subjects. B, Δ z-values for accuracy and certainty for overlapping coherences of the attended and the unattended condition when overall perceptual success rate is the same for the two cueing conditions. A, **B**, means ± standard errors of the mean.

A final concern raised during the course of the review process was addressed in a control experiment. Since the overall success rate was different for attended as compared to unattended trials, during the experiments subjects might have learned to associate invalid cueing with low confidence. In a new experiment six subjects completed a total of 6000 trials in a modified version of the spatial attention task with numerical certainty ratings. Unlike in the original task we presented different coherence levels for the two cueing conditions, on average higher coherences were presented for invalid cues. Coherence levels were chosen as to attain equal or slightly better on average performance in the non-attended compared to the attended condition for each individual. Across all subjects and trials, mean coherence for valid cues was 31.5%, for invalid cues 45.1%. As illustrated in Fig. 5, mean proportion correct for valid cues was 0.76 vs. 0.80 for invalid cues (p = 0.066). Paired differences for z-standardized objective and subjective performance measures for coherence levels presented in both the valid and the invalid condition were extracted and again compared in a final paired t-test: z-standardized increases in certainty (mean 0.89±0.15) with attention were significantly higher (p<0.001) than in accuracy (mean 0.21±0.08). Thus, correcting for the possible confound of the overall success rate, we still observed a significantly stronger influence of voluntary top-down attention on subjective performance measures than on objective performance.

### Feature-based attention increases choice certainty

We observed similar results for the feature-based attention tasks which required subjects to attend to one of two interlacing RDKs differing in color (Fig. 2A). Subjects maintained fixation while one dotfield consisting of an equal number of green and red elements was presented randomly left or right of the fixation point. Color changes of the fixation point to red or green before and after the presentation of this test stimulus instructed subjects which dot elements to direct attention to and which to actually indicate the direction of global motion for. Valid cueing – as defined by matching colors of the precue and the postcue – again was applied in 80% of trials. Subjects signaled post-decision certainty as per the two experimental variations applied in the spatial attention tasks (feature-based attention with either numerical certainty ratings, *FN* in Fig. 2A, or certainty wager, *FW* in Fig. 2A).

Plotted and fitted following the conventions of the two previous tasks, accuracy and certainty again increased for higher motion coherences and with attention, as the shape of the psychometric fits and their leftward shifts for valid trials show (Fig. 2 B–C, F–G). With higher levels of motion coherence, psychometric functions did not approximate maximal accuracy or confidence values, reflecting higher task difficulty due to augmented noise by interlacing RDKs. Increases in accuracy and certainty with attention were significant by means of Monte-Carlo (FN, FW, all p<0.001) and z-statistics (Fig. 3; FN, FW, all p<0.001), irrespective of successful motion discrimination. Within the valid cueing condition accuracy and certainty were significantly different (FN, FW: p<0.001; Fig. 2D, H), within the invalid condition they were for FN (p = 0.018, Fig. 2E) and were not for FW (p = 0.320, Fig. 2I). Logistic fits of accuracy and certainty in FW did not differ significantly by model comparison statistics in the invalid (Fig. 2I), but did for the valid condition (Fig. 2H) with a stronger leftward shift of certainty, again indicating the dissimilar effects of selective attention on accuracy and certainty. All comparisons of z-transformed objective and subjective performance measures performed for the spatial attention tasks were repeated for the feature-based tasks and were highly significant (FN, FW: all p<0.001), again including a separate analysis of correct and incorrect choices (Fig. 3). Attending to the required feature increased certainty significantly more than accuracy for FN (p = 0.009) and tended to do so in FW (p = 0.096; grey comparisons in Fig. 3).

For the feature-based attention task, too, we observed an equal distribution of low and high certainty ratings. See results of paired *t*-tests for valid-cue trials in Fig. 4C (grey brackets/lettering), showing a Gaussian-like distribution of choice categories. For invalid-cue-trials in this task the certainty threshold could not be determined: due to task difficulty for some observers even 100% coherence did not get at a certainty index of 0.625 for unattended trials. Tested for possible effects of outliers, results did not differ considerably. For FN ∼1,3%, for FW ∼0.3% of mean accuracy-certainty pairs per coherence level exceeded a modified z-score of 3.5 and were excluded from a rerun of the z-statitistics. If anything, exclusion of outliers further increased levels of significance without changing the overall picture: FN from p = 0.009 to p<0.001 and FW from p = 0.096 to p = 0.072. As in the spatial attention paradigm, model comparison and z-statistics were repeated without the 25% congruent trials and none of the significant contrasts changed (Δ z-values accuracy vs. certainty comparisons adjusted to p = 0.015 from p = 0.009 in FN).

## Discussion

Whether attention alters our subjective impression of perception has been a subject of theoretical consideration since the pioneering days of experimental psychology [28], [29]. It was not until recently though that a series of experiments demonstrated attentional modulation of phenomenological appearance for various aspects of visual perception [14], [15], [16]. The degree to which a decision-maker believes a choice is likely to be correct is a subjective estimate empirically accessible for instance via numerical confidence ratings or operationalized concepts such as post-decision wagering [1]. Our experimental design allowed simultaneous measurement of the effects of attention on both accuracy and post-decision certainty. Together our findings demonstrate that choice certainty significantly increases with selective attention and this increase is significantly larger than the one observed for accuracy. In other words, the increase in certainty with attention is not reducible to the increase in choice accuracy, or vice versa, but there are independent effects of attention on each performance measure. We show for the first time that spatial (SN, SW) and feature-based attention (FN) affect subjective more than objective performance measures. Our data show this dissociation using manipulations of both spatial and feature-based attention, independent of whether subjective confidence has been indicated by numerical certainty ratings or post-decision wagering, a procedure considered to be more intuitive and objective as subjects are not required to introspect [19], [30]. Respective measures dissociated consistently for 3 out of the 4 different variations of our experiment, arguing for a universal effect of selective attention observed here. The specific changes in choice certainty due to attention did not significantly surpass those seen for choice accuracy in one of the 4 experimental variations (FW). FW included the smallest number of trials in our experimental series and possibly missed significance (p = 0.096) due to a lack of statistical power and small effect size. Notably, certainty in FW – like in the three other experimental variations – increased irrespective of whether perceptual responses were correct or wrong (Fig. 3): in all 4 experiments certainty changes were at least partially independent of changes in objective visual performance. Further studies need to address methodological aspects systematically, e.g. changes in certainty were more prominent for numerical ratings as compared to wagering (Fig. 3).

A recent experiment [17] cued spatial attention in an orientation discrimination task requiring subjects to respond „as fast and as accurate as possible''. Only correct answers were analyzed, and there was no evident difference between certainty for attended and unattended trials. We propose that the speeded response design may have biased the certainty report, conceivably by forcing decision makers to answer as soon as a minimum level of certainty had been attained or as early as they were ready to give an assessment of their confidence in a decision at all. While response times therefore varied with stimulus properties and response accuracy or attention condition, the level of certainty remained rather unchanged. In our series of experiments correct and incorrect trials were examined, and there was no speeded response required. The paradigms and analyses we outlined here allowed to test human observers' confidence without imposed temporal constraints, as post-decision certainty might evolve progressively – even beyond a point in time at which subjects readily release an early response.

Selective attention is a pervasive example of perceptual optimization by appropriately solving inference problems [13], [31] or by addressing resource constraints [14]. Why would attention – the pivotal selection process of the brain – „bother'' to change the degree of confidence? A possible answer is offered by regarding perceptual decision making as a process of probabilistic inference under the terms of Bayes' theorem [32], [33]. Evaluating uncertainty is a necessary first step in a statistical decision. To optimize behavior as per Bayes' rule, subjective certainties reflecting non-dichotomous conditional probabilities [1] of both prior knowledge and sensory input determine their optimal weighting in every perceptual decision [34], [35]. Perceptual optimization through attention might thus come about by refining prior knowledge: choice certainty adjusts the rate of perceptual learning [36]. In addition, the direct influence of attention on certainty may facilitate motor execution [37] by carrying the selection process to the finish.

It is not clear whether the degree of certainty as determined in our experiments might be based on similar neural signals as recorded in behaving rats [2] and monkeys [1]. Representations of both choice and certainty in the lateral intraparietal area (LIP) have been shown in rhesus monkeys for a motion discrimination task using post-decision wagering [1]. Beyond that, LIP encodes signals of attentional selection [11], [12], acting as an integrator that binds visuospatial, motor, and cognitive information into a signal of behavioral salience [38]. In our present study, voluntary top-down attention increased subjective performance measures significantly stronger than objective performance, suggesting at least partially independent mechanisms for choice and confidence formation. These results argue for a modification of the neurodynamical model of gradual accumulation of evidence for both decision and choice certainty in one and the same neural population [1]. The exemplary dissociation of accuracy and certainty rather proposes an implementation by different mechanisms, differing neuronal substrates or in a larger network [2].

The opportunity to disentangle objective and subjective measures in perceptual decision making via selective attention promises to further elucidate the mechanisms representing the formation of a decision and the confidence in it. Both objective and subjective performance measures are to be integrated into attention research, just as manipulations of attention are to be included in the experimental exploration of the neural mechanisms of choice and certainty formation. Offering insights into how the confidence in a decision is implemented in the brain further elucidates the mechanisms underlying perception and learning, and may shed new light on the origins of common psychiatric syndromes involving distortions of perceptual certainty like schizophrenia or obsessive-compulsive disorder.
